# Surface Analysis and Spectrophotometric Evaluation of Different Esthetic Restorative Materials Frequently Exposed to a Desensitizing Agent

**DOI:** 10.1155/2021/9989747

**Published:** 2021-04-30

**Authors:** Neven S. Aref, Reham M. Abdallah

**Affiliations:** ^1^Dental Biomaterials Department, Faculty of Dentistry, Mansoura University, Mansoura, Egypt; ^2^Basic Oral and Medical Sciences Department, College of Dentistry, Qassim University, Buraydah, Qassim, Saudi Arabia; ^3^Dental Biomaterials Department, Faculty of Dentistry, Horus University, New Damietta, Egypt

## Abstract

**Background:**

Patients with tooth sensitivity are frequently exposed to desensitizing agents on a regular basis. These agents might have an impact on the surface properties and color of existing oral restorations. Accordingly, this study aimed to investigate the color stability, surface microhardness, and surface roughness of resin-modified glass ionomer (RMGIC), amalgomer CR, nanohybrid, and bulk-fill resin composites restorative materials after frequent exposure to a desensitizing agent. Materials and Methods. One hundred and twenty specimens were prepared; 10 specimens for each restorative material were equally subdivided into control and desensitizing-agent-exposed groups in each test. Surface microhardness and surface roughness were evaluated using the Vickers microhardness tester and surface profilometer, respectively. The color change was measured by using a spectrophotometer using the CIE *L*^*∗*^*a*^*∗*^*b*^*∗*^ formula. Surface topography was analyzed using a scanning electron microscope (SEM). The collected data were analyzed with Student's *t*-test, one-way ANOVA, and Tukey post hoc tests for pairwise comparison at a level of significance of 0.05.

**Result:**

The frequent use of a desensitizing agent significantly decreased surface hardness of RMGIC, amalgomer, and bulk-fill composite materials. However, nanohybrid composite exhibited a significant surface hardness increase. The surface roughness of RMGIC, amalgomer, and nanohybrid composite increased significantly. Meanwhile, the bulk-fill resin composite showed a nonsignificant decrease. Both RMGIC and amalgomer exhibited significantly higher values of color change in comparison to those of nanohybrid and bulk-fill composites.

**Conclusion:**

The bulk-fill composite seems to be more resistant to discoloration and surface topographical changes than other tested materials on frequent exposure to the desensitizing agent. However, this exposure may pose a negative impact on its surface hardness. Bulk-fill resin composite may be the most suitable esthetic restorative in patients who frequently use desensitizing agents.

## 1. Introduction

Direct restorative materials are commonly used to reconstruct the tooth structure destructed by trauma or dental caries [1]. Ten to twenty years ago, noteworthy progress in restorative materials was recognized in dentistry [2, 3]. Esthetic considerations are growing in importance even for the posterior teeth restorations [4]. For example, resin composite, compomers, glass ionomers (GIs), and resin-modified glass ionomers (RMGIs) have been used as esthetic substitutes for amalgam restorations. These materials fulfilled the patient satisfaction not only for their desirable esthetic but also due to their acceptable physical and chemical properties reflected in the durability of the restoration [3, 4].

Glass ionomers are materials of choice in some patients, particularly those with high caries risk. The chemical bond to the tooth structure and fluoride release responsible for its remineralizing ability are the main advantages of this category [5–7]. However, modifications were necessary to overcome the shortcomings of the conventional ionomer. Among these trials was the development of resin-modified GIC with the additional pros including chemical and micromechanical mechanisms, increased working time, decreased setting time, ease of handling, and improved physical properties and esthetic [8].

A new ceramic-reinforced glass ionomer, amalgomer CR, has been introduced to the dental market. It is a tooth-colored restorative material sponsored by the manufacturer that combines both high mechanical properties of a metallic restorative and the pros of glass ionomers including esthetic [9].

Nanohybrid resin composite has become trendy because it mingles the physical, mechanical, and esthetic properties. It incorporates a high-volume fraction of filler particles with a wide particle size distribution (5–100 nm). The compressive, diametral strength, and the fracture resistance of the nanohybrid resin composite are equivalent to or higher than those of other composites (hybrid, microhybrid, and microfilled-resin composite) [9].

Bulk-fill composite has been introduced as an amalgam substitute. It has a stiffer consistency than conventional hybrid composite, which is produced by altering the particle size distribution or filler type [10]. For some, this stiffer consistency allowed for improved handling characteristics. Another potential advantage of these materials is greater ease in establishing interproximal contacts while placing class II restorations. Additionally, for the rationale of saving time, it could be placed in a thickness of 4 mm and cured in one step as one increment. Consequently, less polymerization shrinkage was reported with those materials compared to the conventional resin-based composites [10]. 

Desensitizing agents have been offered as an approach to overcome the sensitivity accompanied by conservative treatment [11], tooth whitening procedures [12], orthodontic therapy [13], gingival recession, bruxism, and erosion [14]. The usage of these agents in treatment is classified as active treatment of sensitivity [15]. These agents are used to control moderate-to-severe sensitivity. Their mechanism of action depends on either dentinal tubule occlusion or nerve depolarization. For example, tooth whitening agents increase the dentinal pressure within the tubules causing increased dentinal fluid flow with activation of sensory nerve endings and development of pain sensation [16].

On frequent application of desensitizing agents at home by the nonskilled patient, the surface of existing esthetic restorations in the patient's mouth could be accidentally exposed to them. Accordingly, this study aims to decide whether this exposure could influence the surface properties and color stability of these restorations or not. The null hypothesis tested was that the frequent exposure of different esthetic restorative materials to a desensitizing agent would neither influence their surface properties nor color stability.

## 2. Materials and Methods

Four different direct esthetic restorative materials (I, II, III, and IV) were used in the study; resin-modified glass ionomer cement (RMGIC) (Fuji II LC, GC Corporation, Tokyo, Japan) (I), amalgomer CR (Advanced Health Care, Ltd., Tonbridge, UK, Lot no. 071724-3) (II), nanohybrid resin composite (Tetric N-Ceram Ivoclar Vivadent, Schaan, Liechtenstein) (III), and high-viscosity bulk-fill resin composite (Tetric N-Ceram Ivoclar Vivadent, Schaan, Liechtenstein) (IV). A desensitizing agent (Biorepair; Coswell Innovatori Italiano, Funo di Argelato, Italy) has been used.

### 2.1. Experimental Design and Specimen Preparation

A total of 120 specimens were prepared. Three tests were carried out: surface hardness, surface roughness, and color stability. Forty specimens were specified for each test; ten specimens from each restorative material were equally subdivided into two different subgroups; *a*, control (not exposed to a desensitizing agent) and *b*, exposed to a desensitizing agent. Eight groups were assigned as follows:

  Group Ia: RMGIC (control)
  Group Ib: desensitizing-agent-exposed RMGIC
  Group IIa: amalgomer CR (control)
  Group IIb: desensitizing-agent-exposed amalgomer CR
  Group IIIa: nanohybrid resin composite(control)
  Group IIIb: desensitizing-agent-exposed nanohybrid resin composite
  Group IVa: bulk-fill resin composite (control)
  Group IVb: desensitizing-agent-exposed bulk-fill resin composite

For each test, the ten specimens from each material were used; five specimens remained unchanged (control), and the other five specimens were exposed to a Biorepair desensitizing agent. The desensitizer was applied by a gentle rubbing motion with a sterile cotton pellet 3 min/twice a day for 10 days according to the manufacturer's instructions. The test specimens were kept in distilled water at room temperature between frequent exposures to Biorepair. Representative specimens from each group were examined by using a scanning electron microscope to detect surface topographical changes.

### 2.2. Preparation of the Specimens

All materials were handled, cured, finished, and polished according to the manufacturer's recommendations. A sectional Teflon mold (8 mm diameter × 2 mm thickness) was utilized to prepare disc-shaped specimens used for surface microhardness, surface roughness, and color stability tests.

The mold was first positioned over a glass plate and a Mylar strip (Mylar Strip, SS White Co., Philadelphia, PA, USA). Each material was handled according to their manufacturers' instructions and placed into the mold. Another Mylar strip was placed on the mold, and a second glass plate was then positioned over the mold with a gentle pressure to extrude the excess material and produce a standardized surface finishing. Excess material was carefully removed off by using a surgical blade.

For RMGIC, the capsules were triturated for 10 sec in an amalgamator (Fushion SyG-200, TRIUP International Corporation, Shanghai, China) and injected into the mold. Nanohybrid, bulk-fill resin composites, and amalgomer CR were applied as a bulk inside the mold. Nanohybrid and bulk-fill resin composite specimens were irradiated according to the manufacturer's instructions for 10 sec at 850 mW/cm^2^ light intensity using a Demetron LC curing light (Kerr Corporation, Orange, CA, USA) while amalgomer CR was cured chemically.

### 2.3. Surface Microhardness

The Vickers hardness number (VHN) of each specimen was determined using a microhardness tester (Micromet II, Buehler, Lake Bluff, IL, USA). The recommended load was 200 g with 15 sec dwell time. The mean VHN of five indentations made on the surface of each specimen was obtained and expressed in Kg/mm^2^ [17].

### 2.4. Surface Roughness

Surface roughness was assessed using a surface profilometer (Surftest 211, Mitutoyo, Tokyo, Japan). The surface roughness of each specimen was measured in five different locations over the surface. The surface roughness cutoff value was 0.8 mm, and the stylus' traversing range was 4 mm. The radius of the tracing diamond tip was 5 *μ*m, with a measuring strength of 0.4 g and velocity of 0.5 ms^−1^. The average roughness value (Ra) of the five readings of each specimen was obtained and expressed in *μ*m [18].

### 2.5. Color Stability

Assessment of the color coordinates (*L*^*∗*^*a*^*∗*^*b*^*∗*^) was performed using the Vita Easyshade spectrophotometer (Vita Zahnfabrik H. Rauter GmbH & Co., KG, Bad Sackingen, Germany) according to manufacturer's instructions. Measured CIE *L*^*∗*^, *a*^*∗*^, and *b*^*∗*^ at each point were compared to the control specimens for each material, and color difference (Δ*E*) was calculated through the following equation:

(1) $$\begin{array}{c} \Delta E={\left[{\left(\Delta L^{*}\right)}^{2}+{\left(\Delta a^{*}\right)}^{2}+{\left(\Delta b^{*}\right)}^{2}\right]}^{1/2}\text{,} \end{array}$$

where *L*^*∗*^ is the color value (lightness) and *a*^*∗*^ and *b*^*∗*^ denote chromaticity. A Δ*E* value equal or more than 3.3 was considered a clinically perceptible color change [19].

### 2.6. Scanning Electron Microscopy (SEM)

A scanning electron microscope (JSM-6610; JEOL, Peabody, Massachusetts, USA) was used to examine the surface topography of representative specimens from the different groups at a magnification of ×5000.

The Shapiro–Wilk normality test was used to verify the normality of the data. The collected data were analyzed by statistical analysis software (SPSS 12.0, SPSS, Chicago, Illinois) using Student's *t*-test, ANOVA, and Tukey post hoc tests for pairwise comparison with a significant factor of *α* = 0.05. The sample size was calculated by G∗Power software (version: 3.1.9.7). Based on a review of the literature, the authors hypothesized large effect size (*f* = 0.4). In a one-way ANOVA study, sample sizes of 30 were obtained from each of the 4 groups whose means are to be compared. The total sample of 120 units achieves 96% power to detect differences among the means versus the alternative of equal means using an F test with a 0.0500 significance level. The size of the variation in the means is represented by the effect size *f* = *σm*/*σ*, which is 0.4000.

## 3. Results

**Figure 1:**
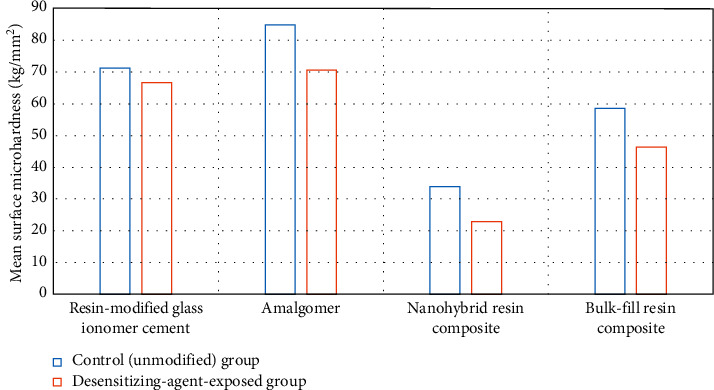
Graphical presentation of Student's *t*-test of surface microhardness (kg/mm^2^) of the tested materials.

**Figure 2:**
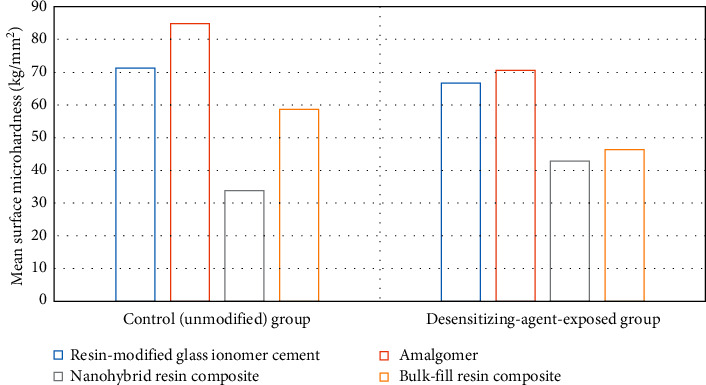
Graphical presentation of ANOVA results of surface microhardness (kg/mm^2^) of the tested materials.

Surface microhardness (kg/mm^2^) results are shown in Table 1 and Figures 1 and 2. For the four restorative materials evaluated, Student's *t*-test indicated that the test group exhibited a significant difference from the control one (*p* ≤ 0.05). Only the desensitizing-agent-treated group of nanohybrid resin composite (IIIb) exhibited a significant increase in the surface microhardness mean value (42.88 ± 1.29) compared to the control group's (IIIa) mean value (33.9 ± 2.72), while other restorative materials, RMGIC, amalgomer, and bulk-fill resin composite test groups (Ib, IIb, and IVb, respectively), showed a significant decrease in their microhardness values (66.66 ± 0.92, 70.59 ± 1.6 and 46.35 ± 2.2, respectively) in comparison with the mean values of their corresponding controls (Ia, IIa, and Iva) (71.26 ± 0.86, 84.85 ± 2.18 and 58.58 ± 2.3, respectively).

On comparing the microhardness of the different restorative materials either before or after the exposure to the desensitizing agent, amalgomer exhibited the highest mean value (84.85 ± 2.18) among controls, and similarly, it had the highest mean value (70.59 ± 1.6) among tested groups of all restorative materials. Conversely, nanohybrid resin composite exhibited the lowest mean value (33.9 ± 2.72) among controls and similarly, it had the lowest mean value (42.88 ± 1.29) among tested groups. One-way ANOVA indicated significance among the different groups (*p* ≤ 0.05). The post hoc test showed that all groups of the tested restorative materials were significantly different from each other either before or after exposure to the desensitizing agent.

**Figure 3:**
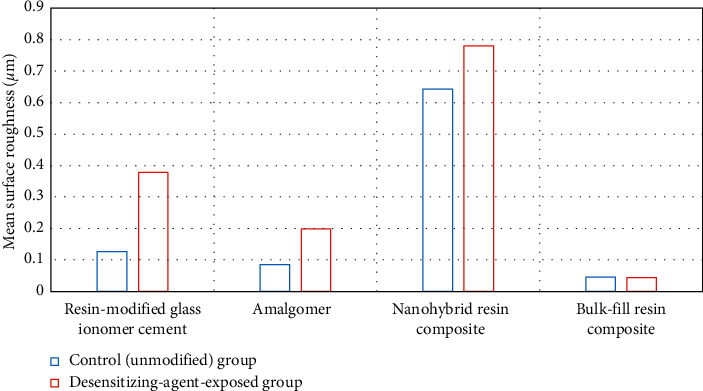
Graphical presentation of Student's *t*-test results of the surface roughness (*μ*m) of the tested materials.

**Figure 4:**
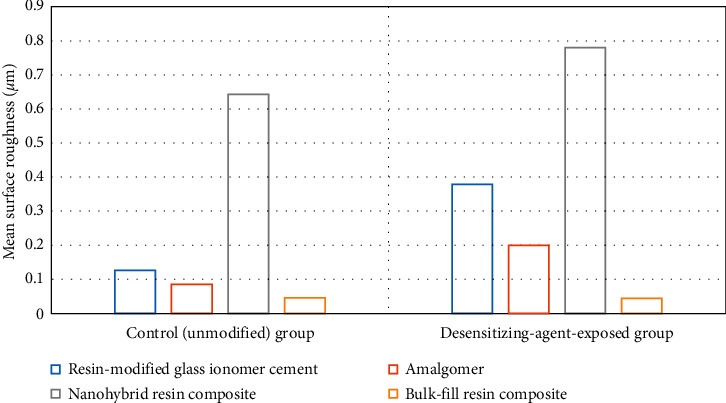
Graphical presentation of ANOVA results of surface roughness (*μ*m) of the tested materials.

Table 2 and Figures 3 and 4 represent the surface roughness (*μ*m) results. Resin-modified glass ionomer cement, amalgomer, and nanohybrid resin composite exhibited a significant increase in their surface roughness on exposure to a desensitizing agent (*p* ≤ 0.05). Solely, bulk-fill resin composite showed a non-significant decrease in the roughness mean value (0.044 ± 0.011) compared to the control group (0.045 + 0.004) (*p* ≥ 0.05).

Using one-way ANOVA for comparison among the different restorative materials either before or after exposure to the desensitizing agent, significance was indicated among groups (*p* ≤ 0.05). Nanohybrid resin composite exhibited the highest mean value (0.643 ± 0.079) among controls, and similarly, it had the highest mean value (0.78 ± 0.064) among tested groups. On the other hand, bulk-fill resin composite exhibited the lowest mean value (0.045 ± 0.004) among controls, and similarly, it had the lowest mean value (0.044 ± 0.011) among tested groups. Among controls, the post hoc test revealed a significant difference between nanohybrid resin composite and RMGIC; nanohybrid resin composite and amalgomer; nanohybrid and bulk-fill resin composites; and RMGIC and bulk-fill resin composite. Conversely, a non-significant difference was found between amalgomer and RMGIC and between amalgomer and bulk-fill resin composite (*p* ≥ 0.05). For the test groups, all groups were significantly different from each other (*p* ≤ 0.05).

**Figure 5:**
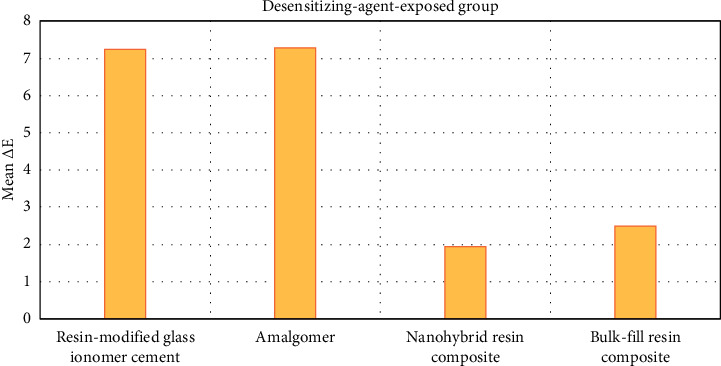
Graphical presentation of the color changes in desensitizing-agent-exposed groups of the tested materials.

Color stability test results are shown in Table 3 and Figure 5. Comparing the color of the different restorative materials after exposure to the desensitizing agent depends on its Δ*E* value. Both RMGIC and amalgomer exhibited high mean values of Δ*E* (7.24 ± 0.32 and 7.28 ± 0.7, respectively) representing much considerable color change. On the other hand, nanohybrid and bulk-fill resin composites demonstrated lower mean values (1.94 ± 0.63 and 2.49 ± 1.07, respectively) indicating a nonperceivable color change.

**Figure 6:**
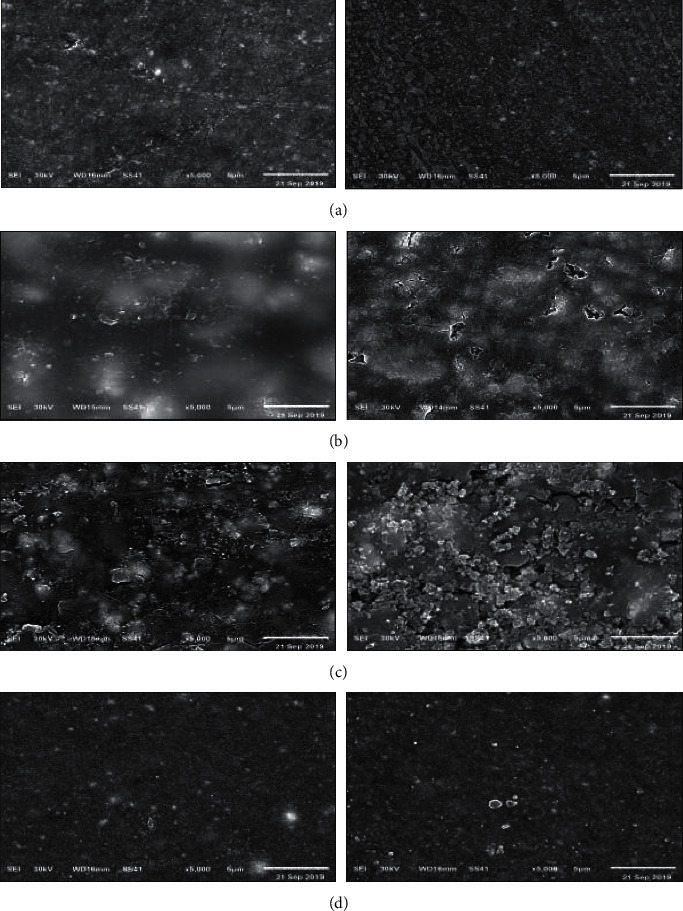
Representative scanning electron micrographs of the used materials: (a) nanohybrid, (b) RMGI, (c) amalgomer, and (d) bulk-fill. Images were visible at baseline (1) and after desensitizing agent application (2).

The SEM images are presented in Figure 6. The scanning electron micrographs showed that there was a loss of homogeneity and smoothness of the surfaces of nanohybrid, RMGI, and amalgomer after the application of the desensitizing agent (Figure 6; (a1 and a2), (b1 and b2), (c1 and c2)), respectively. Some shallow and short microcracks were observed on the surface of RMGIC (Figure 6; b2), while deeper and continuous microcracks were observed on the surface of amalgomer after desensitizing agent application (Figure 6; c2). Randomly distributed small cavities were noted only on the amalgomer surface (6; c2). Bulk-fill images showed a non-significant change in its surface homogeneity after the application of the desensitizing agent (Figure 6; d1 & d2).

## 4. Discussion

In the present study, the null hypothesis was mostly rejected. For the surface microhardness, all the restorative materials have been influenced by the frequent exposure to the desensitizing agent. They all exhibited a significant decrease in their surface hardness values after frequent exposure to the desensitizing agent. The only exception was that of nanohybrid resin composite which showed a significant increase in its surface hardness value. Yet, its surface hardness value is still the lowest one among all of the tested restorative materials before and after exposure to the desensitizing agent. This may be related to the finer filler particle size with less interparticle spacing, more resin matrix protection, and less filler plucking of nanohybrid composite [20, 21]. Furthermore, the nanohybrid resin composite exhibited large clusters formed by the small filler particles that may have aggregated with precipitated calcium and phosphate ions from the desensitizing agent's zinc hydroxyapatite layer and become entangled within the resin components. As a consequence, the composite's three-dimensional microstructure can be enhanced, as well as its surface hardness and resistance to scratching and abrasion [22].

Although amalgomer showed the highest hardness value among the tested materials before and after exposure to the desensitizing agent, yet its hardness value exhibited a significant decrease after exposure to the desensitizing agent. This could be related to the weak bond between zirconia fillers and the glass ionomer matrix, which could facilitate ion penetration from the desensitizing agent's zinc hydroxyapatite layer into the matrix, potentially weakening it [23].

In the current study, SE photomicrographs revealed loss of homogeneity and smoothness in the surfaces of nanohybrid, RMGIC, and amalgomer after the application of the desensitizing agent. On the other hand, bulk-fill micrographs showed a non-noticeable change in its surface homogeneity after the application of the desensitizing agent.

The scanning electron micrographs analysis supports the surface roughness results, in which there was a significant increase in surface roughness values of all RMGIC, amalgomer, and nanohybrid resin composites after their exposure to the desensitizing agent. Meanwhile, the bulk-fill composite exhibited a non-significant decrease in its value. These results can be justified by the fact that the desensitizing agent contains hydrated silica in its composition which possesses an abrasive action on these restorative materials with a corresponding increase in their surface roughness [24]. This finding conforms with the work of Aguiar et al., who postulated that the abrasive nature of hydrated silica in a desensitizing toothpaste increased dentine surface roughness [24].

Moreover, the increased surface roughness may be attributed to the composition of each filling material. For example, the loosely attached zirconia filler in the amalgomer's glass ionomer matrix encourages the loosely attached clustering of calcium and phosphate ions precipitated from the desensitizing agent in the interspaces between them, resulting in an increase in surface roughness [25]. The organic resin matrix within the nanohybrid resin composite also contributes to the increased surface roughness. Monomers used in nanohybrid composites, such as bis-GMA and ethoxylated bisphenol-A dimethacrylate (Bis EMA), have lower cyclization, more cross linking, and higher stiffness in the polymer, which interfere with the penetration and homogenization of the precipitated calcium phosphate layer from the desensitizing agent into the nanohybrid composite surface [26]. This calcium phosphate layer may remain attached to the surface with nonuniform distribution causing an increase in the surface roughness. Additionally, this explanation may be supported by the SE micrograph analysis and surface hardness results of the nanohybrid resin composite, in which this layer may participate in the increase of the surface hardness value of this composite after exposure to the desensitizing agent.

As for RMGIC, the precipitating ions from the desensitizing element may pose an abrasive or erosive role on its resin matrix with a resultant protrusion of its fillers that may, in turn, increase the corresponding surface roughness. This finding is in line with that of Oya et al., who discovered that RMGIC with smaller particle sizes becomes rougher after exposure to abrasive and erosive materials than traditional glass ionomer cement [27]. Conversely, the lower surface roughness of the bulk-fill composite is due to a higher filler content with finer particle size and higher viscosity, which decreases the precipitating ions from the desensitizing element's impact on the bulk-fill surface. This finding is consistent with the findings of many studies [28, 29].

The importance of small color variations and their limits in terms of perceptibility and acceptability is paramount [30, 31]. While data on acceptability and perceptibility limits are conflicting, a Δ*E*^*∗*^ value of 1 was found to be undetectable by 50% of observers [32]. A Δ*E*^*∗*^ value of 2 or less was found to be within reasonable clinical limits [33]; the 50 : 50 Δ*E*^*∗*^ replacement point of esthetic dental products was 2.7 [34], whereas a Δ*E*^*∗*^ of 3.3 was found to be visually undesirable discoloration and was used as a gold standard by most researchers [19].

Both RMGI and amalgomer exhibited higher significant values of color change in comparison to those of nanohybrid and bulk-fill resin composites representing a much perceptible degree of color change. This conforms to the results of surface roughness of both RMGIC and amalgomer by the effect of hydrated silica abrasiveness on them [24]. These findings agree with those of Yu et al., who reported that surface roughness caused by wear and abrasion can affect gloss and, as a result, increase the likelihood of discoloration of restorative materials [35].

Meanwhile, a nanohybrid composite resin with smaller particle size is expected to have a smoother surface and achieve greater color consistency. Furthermore, nano-sized fillers will fill the interparticle spaces in the material, resulting in increased surface resistance to the abrasive effect of hydrated silica, which can compromise surface color stability [36].

This result was supported by a previous study [37], which found that nanofilled resin composite was more color stable than microhybrid composite resins. However, another study found that the nanofilled composite resin changed color more than the microhybrid composite resin after immersion in various beverages [36].

In the case of bulk-fill composites, higher filler content with finer particle size and higher viscosity accounts for their resistance to hydrated silica abrasiveness, resulting in lower surface roughness and color shift values [38].

The present study tested the effect of the desensitizing agent on different mechanical and optical properties of restorative materials. It would be interesting in the future to test also remineralizing agents, such as biomimetic hydroxyapatite [39] or casein phosphopeptide-amorphous calcium phosphate [40], in order to increase the knowledge about dental material characteristics.

The prior studies that are relevant to this study and provide the theoretical foundations for the research question we are investigating might be limited. Moreover, water sorption and solubility tests, as well as atomic force microscopy analysis, might be conducted to support the findings of this study. The research may also be applied to a variety of restorative dental materials, such as traditional glass ionomer cement, and several forms of desensitizing agents.

## 5. Conclusions

Based on the results and within the limitations of this study, it could be concluded that the frequent exposure of RMGIC, amalgomer, and bulk-fill materials to desensitizing agents decreased their surface hardness significantly. However, nanohybrid composite resin exhibited a significant increase in the surface hardness value. The surface roughness values of all RMGIC, amalgomer, and nanohybrid resin composites increased significantly after their exposure to the desensitizing agent. Meanwhile, the bulk-fill composite showed a non-significant decrease in its value. Both RMGIC and amalgomer exhibited higher significant values of color change in comparison to those of nanohybrid and bulk-fill resin composites representing a much perceptible degree of color change. Bulk-fill resin composite may be the most appropriate choice of esthetic restorative material for patients who frequently use desensitizing agents as it is the only one that exhibited comparable or even less surface roughness with a clinically agreeable color change on exposure to the desensitizing agent. As well, its diminished surface hardness, still higher than the increased value of the nanohybrid resin composite frequently exposed to the tooth desensitizer.

## Figures and Tables

**Table 1:** Student's *t*-test, ANOVA, and post hoc tests of the surface microhardness results.

Group	Control		Tested		*t* value	*p* value
	Mean	SD	Mean	SD		
RMGIC	71.26^*b*^	0.86	66.66^*b*^	0.91	8.17	0.0001
Amalgomer	84.85^*a*^	2.18	70.59^*a*^	1.6	11.78	0.0001
Nanohybrid	33.9^*d*^	2.72	42.88^*d*^	1.29	6.66	0.0002
Bulk-fill	58.58^*c*^	2.33	46.35^*c*^	2.25	8.45	0.0001
LSD value	0.0786		2.1354		—	—
*p* value	0.0001		0.0001		—	—

LSD; least significant difference. *a*–*d*; means with the same letter in each column are not significantly different at *p* ≤ 0.05.

**Table 2:** Student's *t*-test, ANOVA, and post hoc tests of the surface roughness results.

Group	Control		Tested		*t* value	*p* value
	Mean	SD	Mean	SD		
RMGIC	0.126^*b*^	0.012	0.379^*b*^	0.095	5.93	0.0004
Amalgomer	0.085^*bc*^	0.010	0.199^*c*^	0.025	9.65	0.0001
Nanohybrid	0.643^*a*^	0.079	0.780^*a*^	0.064	3.02	0.0166
Bulk-fill	0.045^***c***^	0.004	0.044^*b*^	0.011	0.19	0.8571
LSD value	0.0543		2.8694		—	—
*p* value	0.0001		0.0001		—	—

LSD; least significant difference. *a*–*d*; means with the same letter in each column are not significantly different at *p* ≤ 0.05.

**Table 3:** ANOVA and post hoc tests of the color difference results for the different restorative materials.

Test group	Mean ± SD	*f* value	*p* value
RMGIC (Ib)	7.24 ± 0.32^*a*^	79.41	<0.0001
Amalgomer CR (IIb)	7.28 ± 0.7^*a*^		
Nanohybrid resin composite (IIIb)	1.94 ± 0.63^*b*^		
Bulk-fill resin composite (IVb)	2.49 ± 1.07^*b*^		

Means with the different superscript letters are significantly different at *p* ≤ 0.05.

## Data Availability

All data presented or analyzed during this study are included within this article.
